# Supplementary material for: Mentha spp. Essential Oils: A Potential Toxic Fumigant with Inhibition of Acetylcholinesterase Activity on Reticulitermes dabieshanensis
Source: Plants (Basel). 2023 Nov 30;12(23):4034. doi: 10.3390/plants12234034 (PMC10708454; doi:10.3390/plants12234034)
Supplement: Supplementary file 1 [file plants-12-04034-s001.zip › plants-2679894-supplementary.pdf]

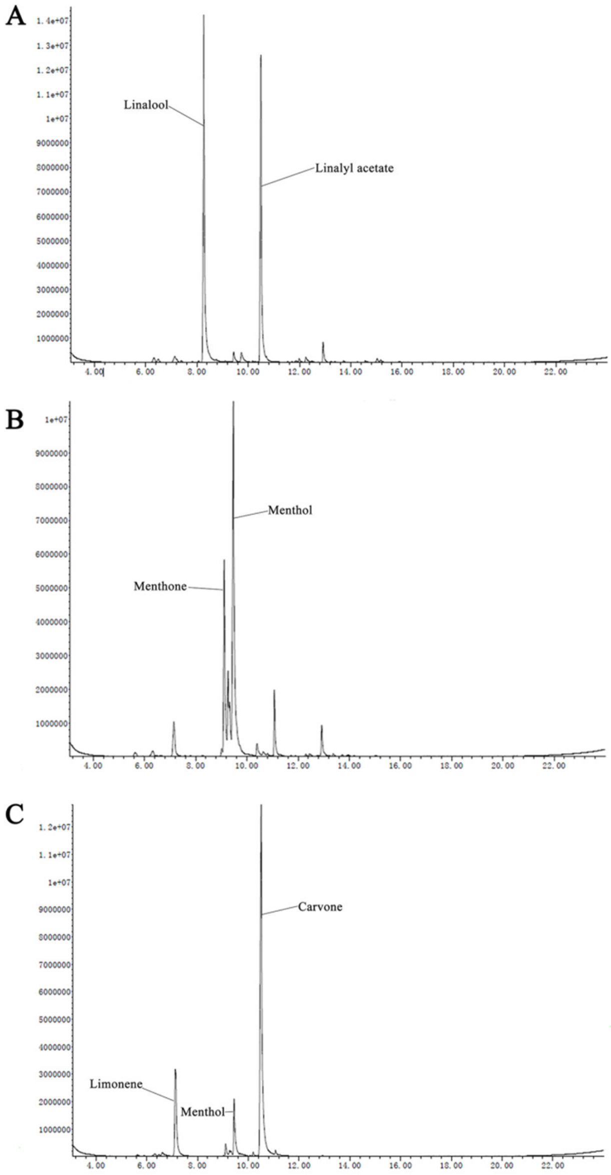

Figure S1. Gas chromatogram of the essential oils from *Mentha citrata* (A), *Mentha Piperita* (B) and *Mentha spicata* (C).
